# Single-Step Production of a Recyclable Nanobiocatalyst for Organophosphate Pesticides Biodegradation Using Functionalized Bacterial Magnetosomes

**DOI:** 10.1371/journal.pone.0021442

**Published:** 2011-06-28

**Authors:** Nicolas Ginet, Romain Pardoux, Géraldine Adryanczyk, Daniel Garcia, Catherine Brutesco, David Pignol

**Affiliations:** 1 CEA, DSV, IBEB, Laboratoire de Bioénergétique Cellulaire, Saint-Paul-lez-Durance, France; 2 CEA, DSV, IBEB, Laboratoire des Interactions Protéines Métal, Saint-Paul-lez-Durance, France; 3 CNRS, UMR 6191 Biologie Végétale & Microbiologie Environnementale, Saint-Paul-lez-Durance, France; 4 Aix-Marseille Université, Saint-Paul-lez-Durance, France; University Paris Diderot-Paris 7, France

## Abstract

Enzymes are versatile catalysts in laboratories and on an industrial scale; improving their immobilization would be beneficial to broadening their applicability and ensuring their (re)use. Lipid-coated nano-magnets produced by magnetotactic bacteria are suitable for a universally applicable single-step method of enzyme immobilization. By genetically functionalizing the membrane surrounding these magnetite particles with a phosphohydrolase, we engineered an easy-to-purify, robust and recyclable biocatalyst to degrade ethyl-paraoxon, a commonly used pesticide. For this, we genetically fused the *opd* gene from *Flavobacterium sp.* ATCC 27551 encoding a paraoxonase to *mamC*, an abundant protein of the magnetosome membrane in *Magnetospirillum magneticum* AMB-1. The MamC protein acts as an anchor for the paraoxonase to the magnetosome surface, thus producing magnetic nanoparticles displaying phosphohydrolase activity. Magnetosomes functionalized with Opd were easily recovered from genetically modified AMB-1 cells: after cellular disruption with a French press, the magnetic nanoparticles are purified using a commercially available magnetic separation system. The catalytic properties of the immobilized Opd were measured on ethyl-paraoxon hydrolysis: they are comparable with the purified enzyme, with *K*_m_ (and *k*_cat_) values of 58 µM (and 178 s^−1^) and 43 µM (and 314 s^−1^) for the immobilized and purified enzyme respectively. The Opd, a metalloenzyme requiring a zinc cofactor, is thus properly matured in AMB-1. The recycling of the functionalized magnetosomes was investigated and their catalytic activity proved to be stable over repeated use for pesticide degradation. In this study, we demonstrate the easy production of functionalized magnetic nanoparticles with suitably genetically modified magnetotactic bacteria that are efficient as a reusable nanobiocatalyst for pesticides bioremediation in contaminated effluents.

## Introduction

The use of enzymes in industrial applications has been limited by several factors, mainly the high cost of the enzymes, their instability, and availability in small amounts. Immobilization of biocatalysts helps in their economic re-use, in the development of continuous bioprocesses and offers the possibility of wider and more economical exploitation of biocatalysts in industry, waste treatment and medicine. Regardless of its nature or preparation, an immobilized enzyme must perform two essential roles: the non-catalytic functions that facilitate its handling (e.g., recovery of the catalysts from the application environment, their re-use for several cycles); and the catalytic functions that aim to convert the targeted substrates within a desired time and space [1]. There is no universally applicable method for enzyme immobilization but it always requires (i) the production of the functional enzyme itself (expression, maturation and purification), (ii) the selection of a suitable carrier and (iii) an efficient method to connect both compounds (such as covalent binding, adsorption, specific affinity interaction). Each of these processes must be separately optimized. Clearly, a single-step biological self-assembly method would represent an important advance in the field, especially if it can be used for membrane-bound or cofactor-dependent enzymes. In recent years, numerous types of magnetic particles (on the order of micrometer or nanometer dimensions) have been used as carriers for protein immobilization in various applications (e.g., environmental remediation, sensing devices, cell labelling, immunomagnetic separations, magnetic resonance imaging, targeted drug delivery, and bio-imaging) [2], [3]. Low-cost magnetic particles are now commercially available and can be functionalized by various methods. Such immobilized bioactive substances can be easily manipulated with a magnetic field; these nano-objects are widely used in life sciences (e.g. cell sorting, enzyme purification, proteome recovery) and biomedical applications (e.g. targeted contrasting agent in Magnetic Resonance Imaging). Nevertheless, grafting of a biologically active enzyme onto magnetic platforms requires multiple steps, and its functionalization with membrane proteins is a challenge that remains to be met satisfactorily. By contrast, the use of a magnetite-producing microorganism to prepare biogenic particles (incorporating both the magnetic carrier and the catalytic compound) in a single step is a promising alternative.

**Figure 1:**
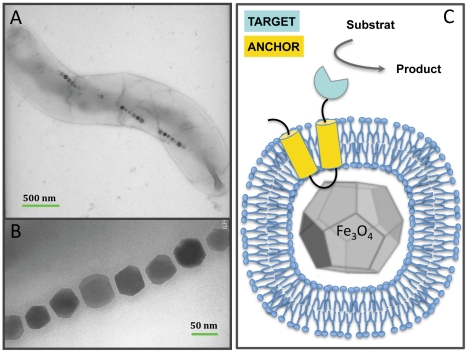
Functionalization of bacterial magnetosomes. (**a, b**) TEM image of magnetosomes in *Magnetospirillum magneticum* AMB-1 cells. The magnetite crystals are aligned within the cytoplasm of the cells on a cytoskeleton made of actin-like proteins. (**c**) Functionalization of the magnetosome membrane. The targeted enzyme (blue) is anchored on the lipid-coated magnetite crystals by fusion with a membrane protein (yellow).

Magnetosomes are nano-sized particles of magnetite (Fe_3_O_4_) or greigite (Fe_3_S_4_) that are biomineralized by a phylogenetically diverse group of prokaryotes, the magnetotactic bacteria (MTBs) [4], [5]. These mono-domain magnetic crystals are embedded in lipid vesicles containing specific proteins. Magnetosomes are regularly aligned within the cytoplasm, forming a rod with a magnetic moment sufficient for the bacteria to align passively along the geomagnetic field lines (Fig. 1A and B). The genetic determinants of this unique behaviour are clustered in a genomic island that has been passed among various groups of prokaryotes by lateral gene transfer [6], [7]. Since their independent discovery by Bellini in 1963 [8], [9] and Blakemore in 1975 [4], magnetosomes aroused interests for biotechnologies and we can found in the scientific literature mentions covering a broad range of applications, such as magnetic resonance imaging, hyperthermia cancer treatment, or molecule and cell sorting. Functional soluble proteins (GFP or luciferase) have been grafted on magnetosome surfaces by chemical coupling of specific ligands to membrane lipids or proteins, or displayed by gene fusion using as an anchor proteins specifically targeted to the magnetosome membrane (such as MagA, Mms16 or MamC) [10] (Fig. 1C). The latter approach circumvents the purification steps, as the enzyme is genetically targeted to the membrane surrounding the crystal: subsequently, the functionalized magnetite particles can be magnetically recovered after a simple disruption of the cells. The production of MTBs can be achieved with a reasonable yield under controlled conditions in biorecactors although improvement of cellular growth of these microaerophilic organisms is an objective shared by different research groups worldwide, including ours. Most recent reports of applied projects involving magnetosomes relate to cell sorting or DNA sequencing [11]–[13]. However, the bioremediation of pollutants from soil and water represents a vast field of biotechnological applications for magnetosomes but still poorly explored. There is an increasing societal demand for inexpensive, robust, environmentally friendly processes that can remediate human-generated pollution. Here, we describe as a proof of concept the single-step production of a reusable magnetic biocatalyst destined for such bioremediation, namely the *in vivo* immobilization of a bacterial phosphotriesterase. This dimeric zinc metalloenzyme is able to hydrolyze organophosphorous neurotoxins such as pesticides (i.e. paraoxon) or some nerve agents; thanks to these catalytic properties this family of enzymes has elicited great interest for the monitoring and bioremediation of polluted environments [14].

## Results

First, we genetically fused the organophosphohydrolase (Opd) of *Flavobacterium sp.* ATCC 27551 [15] with MamC, a protein of unknown function found in abundance specifically in the magnetosome membrane. We thus used the fused MamC to anchor the enzyme onto the cytoplasm-facing surface of the magnetosome as to date this protein proved to be the most efficient for molecular display [16]. The broad-host-range plasmid pBBR1MCS2 bearing the chimerical construct was then introduced into wild-type *Magnetospirillum magneticum* AMB-1, the model magnetotactic bacterium, by biparental mating. Recombinant bacteria were screened for their dual ability to hydrolyze ethyl-paraoxon *in vivo* (by colorimetric titration as described in Materials and Methods) while retaining a magnetotactic behavior (monitored by magneto-spectrophotometry with a lab-modified spectrophotometer [17]). Cells were harvested at the end of the exponential growth phase and disrupted with a French press. After centrifugation, the pellet containing magnetosomes was resuspended and magnetically purified on a column (MiniMACS®, Miltenyi Biotec). After 3 washing steps, purified functionalized magnetosomes were eluted by flow gravity simply by removing the magnetic field. The quality and the homogeneity of the crystals thus obtained were checked by TEM.

**Figure 2:**
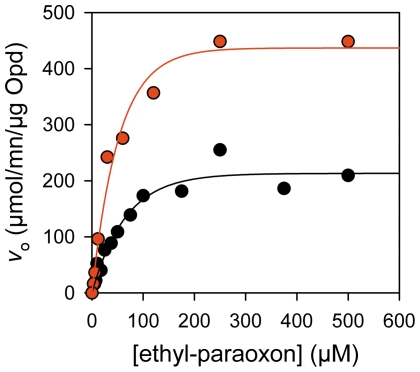
Catalytic properties of MamC-Opd in the magnetosomes sample. Michaelis-Menten curves for immobilized (black) and purified (red) Opd. For each enzymatic assays, we used 26 ng of immobilized Opd (20 µl of magnetosomes suspension) and 200 ng of soluble Opd. The mean deviation between the fitted Michaelis-Menten curves (lines) and the experimental data points (circles) is 4.2% for both curves.

**Figure 3:**
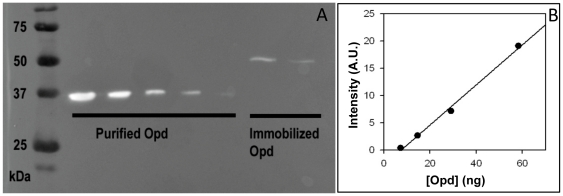
MamC-Opd assay in the magnetosome sample. (a) Western blot assay of purified and immobilized Opd. The purified protein deposits are 117, 58, 29, 15 and 7 ng, from left to right. The magnetosome membrane was dissolved in 4% SDS and two deposits of solubilized membrane proteins were made (15 and 10 µl). Expected molecular weights for Opd and MamC-Opd are 34.6 and 49.6 kDa respectively. (b) Calibration curve for Opd quantification obtained with GeneTools image analysis software from Syngene. The computed Opd quantities in the solubilized magnetosome membrane proteins are 17.3 and 9.6 ng for the 15 and 10 µl of solubilized magnetosome membrane protein deposits (from left to right).

Next, we investigated the preservation of the catalytic function on the magnetic bio-nanoparticles, its stability and the catalyst recovery. We used ethyl-paraoxon as a model organophosphate to investigate the catalytic activity as its hydrolysis can be easily monitored by spectrophotometry. We found that the catalytic properties of the Opd immobilized on the magnetosome surface are conserved with respect to the soluble enzyme we purified. Fig. 2 summarizes the spectrophotometric assays of the hydrolase activity monitoring the *p*-nitrophenol release at 401 nm due to ethyl-paraoxon hydrolysis by Opd. The Michaelis-Menten plot shows that the affinity for the substrate is not affected by the immobilization: the *K*_m_ values are 58.0±2.5 µM and 43.0±1.8 µM for the immobilized and purified Opd respectively. Furthermore, we quantified the immobilized enzyme with an antibody raised against Opd (see Western blot displayed in Fig. 3). In the 15 and 10 µl deposits of solubilized magnetosome membrane proteins, the calculated quantities are 17.3 and 9.6 ng of immobilized Opd respectively. Thus the average value in the solubilized sample is 1.05 ng.µl^−1^. Thus the quantity of immobilized in the magnetosomes suspension prior to solubilization is 1.3 ng.µl^−1^, i.e. [Opd] = 37.5 nM (34.6 kDa for Opd). Thus the turnover numbers *k*_cat_ are 151±6 s^−1^ vs. 314±13 s^−1^ for the immobilized and purified enzyme. Together, these results demonstrate that i) the zinc cofactor is properly inserted in the metalloprotein by the manufacturing cell, yielding an active holoenzyme; ii) the functional enzyme is displayed at the magnetosome surface and accessible to the substrate; iii) the enzymatic activity of the immobilized enzyme is similar to the purified enzyme.

**Figure 4:**
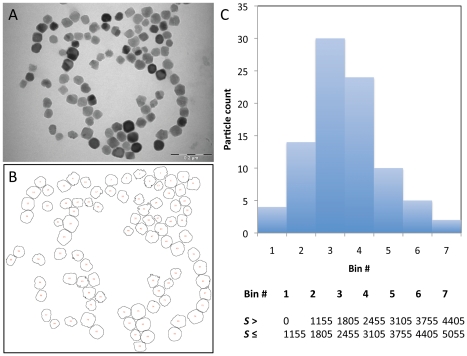
An estimate of the volume of a single particle from TEM images. Scale bar is 0.2 µm. (**a**) Unprocessed TEM image of the functionalized magnetosomes. (**b**) Automatic particle counting and sizing analyzed by ImageJ. (**c**) Particles size distribution computed with 7 bins (boundaries are given in the table). The surface *S* is expressed in pixel^2^.

The efficiency of these functionalized nanoparticles for bioremediation processes is modulated by the catalytic properties of the enzyme but also by the number of enzymes functionally displayed at the surface of the crystals. As we know the concentration of Opd present in our sample, the only missing parameter is [Magn], the magnetosomes concentration. This value can be estimated with the following equation:
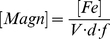
(1)where *f* = 0.7236 is the mass fraction of iron contained in one magnetite molecule (Fe_3_O_4_, MW 231.5 g.mol^−1^), *d* = 5.2.103 g.l^−1^ is the density of magnetite, [Fe] = 2.31 g.l^−1^ is the iron content in the magnetosome suspension we determined by ICP-AES and *V* = is the average volume of a single magnetosome. The latter value can be computed from TEM images treated by the image processing and analysis freeware ImageJ (http://rsbweb.nih.gov/ij/). The raw image obtained in TEM (Fig. 4A) is processed to yield a threshold image and analyzed for particle counting and surface calculation (Fig. 4B). At this stage, we need to simplify the geometry of a single magnetosome as the magnetite nanocrystals crystalline form is cubo-octaedric in *Magnetospirillum magneticum* AMB-1 with a shape factor close to 1 [5]: for simplification we hypothesize a cubic form for the magnetite nano-crystals. Knowing the average surface *S* of a single magnetosome, its volume *V* will thus be:
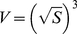
(2)The mean surface *S* estimated from the image presented in Fig. 4B is 3,120 pixel^2^, thus *V* = 174,274 pixels^3^. On the TEM image 264 pixels represent 0.2 µm, i.e. 7.5810^−10^ m.pixel^−1^. Then the average volume of a cubic magnetsome is *V* = 7.59 10^−23^ m^3^. The analysis report generated by ImageJ is summarized in the histogram in Fig. 4C. Using Eq. 1, the magnetosomes concentration [Magn] is 13.5 nM. As we found previously [Opd] = 37.5 nM, the average number of Opd macromolecules displayed on each nanoparticle is 2.8.

**Figure 5:**
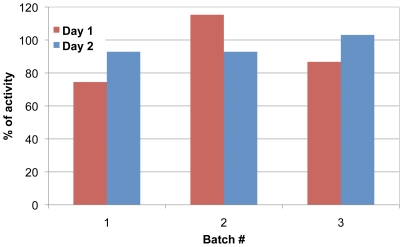
Recycling of the biocatalyst. A 500 µl aliquot of magnetosomes suspension is immobilized onto the magnetic column. A 1 ml sample contaminated with 50 µM ethyl-paraoxon is passed through the column by flow gravity and recovered for *p*-nitrophenol spectrophotometric assay. The column is stored at room temperature with the magnetosomes and the experiment is repeated two more times at 2-hour intervals. Magnetosomes still magnetically retained on the column are stored at 4°C overnight and the entire set of experiments is repeated the next day. The hydrolysis activities are plotted for each repeat (100% activity expresses the complete degradation of ethyl-paraoxon by the purified enzyme) for day 1 (red) and day 2 (blue).

Finally, to assess the stability of the functionalized magnetosomes and their suitability as a reusable catalyst for bioremediation, we immobilized a 500 µl aliquot of magnetosome suspension on the magnetic column. Then a 1 ml sample contaminated with 50 µM ethyl-paraoxon is passed through the column by flow gravity and recovered for *p*-nitrophenol spectrophotometric assay. The column is stored at room temperature with the magnetosomes and the experiment is repeated two more times at 2-hour intervals. Magnetosomes still magnetically retained on the column are stored at 4°C overnight and the entire set of experiments is repeated the next day. The hydrolysis activities are plotted for each repeat (100% activity expresses the complete degradation of ethyl-paraoxon by the purified enzyme) for day 1 (red) and day 2 (blue). As shown in Fig. 5, approximately 80% of the pollutant is hydrolyzed in one passage (compare to 100% for the purified enzyme). In subsequent trials, between 90 and 100% of the ethyl-paraoxon was degraded at each passage on the column. Here, we demonstrate the stability of the biocatalyst over repeated use and short-term storage.

## Discussion

Opd-functionalized magnetosomes can be readily used after extraction from bacteria, whereas traditional methods require multiple steps to graft the enzyme to a suitable carrier, and for enzyme purification. Functional properties are liable to be degraded due to the chemical processes required for enzyme immobilization on the synthetic magnetic nanoparticles. Our experimental design illustrates the ease in which magnetosomes are immobilized and recovered using a commercially available magnetic column system. We will follow two lines of research to optimize the molecular display efficiency on the magnetosome surface. The first project aims to increase the number of Opd displayed (we computed an average number of 2.8 enzyme units per magnetosome) by using *Magnetospirillum magneticum* AMB-1 mutant strains where the chromosomal gene encoding the selected anchor is deleted (MamC in the present work); in addition we can remove from the bacterial chromosome genes encoding other membrane proteins that have been shown to be unessential for magnetosome biogenesis [7]. The second project aims to test other proteins that are found specifically in the magnetosome membrane that could also anchor Opd, thereby increasing the number of chimerical anchor-Opd complexes displayed on the magnetosome surface.

To conclude, the method presented in this work greatly simplifies the production of functionalized magnetic nanoparticles that can be used in bioremediation processes. The catalytic function is preserved, easily recovered and efficiently reused. These features indicate that the stream-lined elegance of our laboratory-based experiment should easily scale up to the demands of field site bioremediation.

## Materials and Methods

### Bacterial cultures

*Magnetospirillum magneticum* AMB-1 wild-type strain was cultured in modified *Magnetospirillum* growth medium [7], supplemented with Wolfe's vitamin solution (1 ml of stock solution per 100 ml of culture medium), 30 µM ferric malate. When needed 10 µg.ml^−1^ kanamycine was added. Cells were grown for 4 days in Schott bottles containing 2 l of growth medium inoculated with 100 ml of pre-culture. Prior to incubation at 30°C, bottles were flushed with 2% oxygen. Heterologous expression of his-tagged Opd was made in *E. coli* BL21. The cells were cultured in flasks containing 3 l of LB medium supplemented with 50 µg.ml^−1^ ampicillin at 37°C, 150 rpm agitation, until O.D. at 600 nm = 0.6 is reached. Protein expression is then induced by the addition of 0.1 mM IPTG and cells grown overnight under agitation (150 rpm) at 37°C.

### Molecular biology

All primers used in this section are listed in Table 1. A truncated version of the *opd* gene, omitting the 87 bp signal sequence, was amplified by PCR from *Flavobacterium* sp. ATCC 27551 [15] genomic DNA (primers Opd-F and Opd-R). The PCR product was cloned into the pET100 vector system from Invitrogen, (providing an in frame 6 histidines tag sequence at the 5′ extremity of the gene), yielding pET100-Opd. The plasmid pMamC-Opd expressing the transcriptional fusion *mamC::opd* is derived from the broad-host expression vector pBBR1MCS-2 bearing the kanamycin resistance cassette [18]. The *mamC* gene was amplified by PCR from *Magnetospirillum magneticum* AMB-1 genomic DNA [19], omitting the stop codon (primers MamC-F and MamC-R) then cloned between EcoRI and XmaI in pBBR1MCS-2, yielding pMamC. The truncated *opd* gene was amplified from pET100-Opd with primers B-Opd-F and S-Opd-R, introducing the Tobacco Tech Virus (TEV) protease cleavage site at the 5′ extremity of the gene (primers B-Opd-F and S-Opd-R). This fragment was then cloned between BamHI and SacI in plasmid pMamC, yielding pMamC-Opd. This plasmid was then transformed into *E. coli* WM3064 electrocompetent cells and then conjugated in wild-type *Magnetospirillum magneticum* AMB-1 bi-parental mating as described elsewhere [7].

**Table 1:** Primers used for the *mamC::opd* transcriptional fusion.

Primer	Sequence	Features
**Opd-F**	5′-CACCATGTCGATCGGCACAGGC-3′	Sequence for cloning in pET100 (underline)
**Opd-R**	5′-**TCA**TGACGCCCGCAAGGTCG-3′	Stop codon (bold)
**MamC-F**	5′-AGAATTCAGGACAACAGCG**ATG**CCCTTTCACCTTGCCCCCTATCTGG-3′	EcoRI restriction site (underline), initiation codon (bold)
**MamC-R**	5′-TATACCCGGG **GCCCTGGAAGTACAGGTTCTC**GGCCAGTTCGTCCCGCAAGATG-3′	XmaI restriction site (underline), TEV protease cleavage site (bold)
**B-Opd-F**	5′-AAAGGATCCTCGATCGGCACAGGC-3′	BamHI restriction site (underline)
**S-Opd-R**	5′-AGAGCTC **TCA**TGACGCCCGCAAGGTCGG-3′	SacI restriction site

### Enzyme purification

Harvested *E. coli* BL21 expressing Opd were resuspended in 50 mM Tris-HCl (pH 8), 250 mM NaCl, then disrupted with a French Press operating at 1,000 Psi. After removing the cell debris, a centrifugation for 45 mn, at 150,000 g yielded soluble proteins that were loaded on a Ni-NTA affinity resin. The His6-tagged protein was eluted by imidazole step gradient (50 mM wash and 250 mM elution). A final gel filtration was performed in a buffer containing 50 mM Hepes (pH 8) and 50 mM NaCl on a Sephadex G200 26/60 (GE Healthcare). The yield of purified Opd was typically of 5 mg per liter of culture (BCA protein assay).

#### Magnetosomes purification

Bacteria were harvested by centrifugation (7,500 g, 10 mn, 4°C) then diluted in 15 ml of 20 mM Hepes buffer (pH 8) supplemented with 5 µl antiproteases cocktail and 5 µl DNAse I. Cells were disrupted 3 times using a French press (1,000 psi). After centrifugation (7,500 g, 5 mn, 4°C), the pellet containing magnetosomes was resuspended 1,5 ml of Hepes buffer. An additional centrifugation was performed (16,000 g, 1 mn, room temperature) and the pellet was diluted in a final volume of 0.9 ml of Hepes buffer. In order to separate magnetosomes from cells debris, the suspension was passed through a magnetic column by flow gravity (MS column for MACS® separation cell, Miltenyi Biotec). This column contains iron filings and is placed in the gap of a magnet; magnetosomes are thus retained on the column. The column was washed 3 times with 1 ml of Hepes buffer. Magnetosomes were eluted with 1 ml of Hepes buffer with the magnetic field removed. A final centrifugation (16,000 g, 1 mn, room temperature) followed by a dilution in 1 ml of Hepes buffer completed the magnetosomes purification.

### Magnetosome proteins extraction

The magnetosome suspension was vigorously vortexed to ensure a homogenous dispersion of the particles. The magnetosome membrane was dissolved by the addition of 80 µl of SDS 20% (w/w) to an aliquot of 320 µl of magnetosomes solution. After 60 mn incubation at room temperature (homogenized every 10 mn by vortexing), the sample was centrifuged to remove the magnetite crystals (16,000 g, 1 mn, 4°C). The supernatant was sampled for BCA protein assay, SDS PAGE and Western Blot analysis. Magnetite crystals were kept for iron assay by ICP-AES.

### Enzyme kinetics

The hydrolase activity of Opd and MamC-Opd was measured by absorption spectroscopy, following at 401 nm the release of *p*-nitrophenol (*e*_401 nm_ = 18,380 M^−1^.cm^−1^) after hydrolysis of ethyl-paraoxon by the enzyme. Either 200 ng of purified Opd or 20 µl of the homogenized magnetosome suspension were added in a 2 ml plastic cuvette containing 1 ml of 25 mM Tris-HCl buffer (pH 8.8), 100 µM ZnCl_2_ (a booster of the enzymatic activity). After 10 mn incubation at 30°C, the reaction was triggered by addition of ethyl-paraoxon from stock solutions in ethanol at 100, 10 and 1 mM. The initial slope of the absorbance increase was recorded for each sample and plotted against the substrate concentration to plot the Michaelis-Menten curve. Data were fitted with SigmaPlot analysis software and *K*_m_ and *V*_m_ parameters were estimated after normalization by the enzyme quantity measured elsewhere.

### MamC-Oph quantification

For immunochemical titration, a range of purified Opd standards (7.7, 3.9, 1.9, 1.0 and 0.5 ng.µl^−1^) were prepared in 20 mM Hepes buffer (pH 8). Prior to SDS-PAGE analysis, 15 µl of each protein samples were supplemented with 5 µl of SDS sample buffer and boiled for 5 mn. The aliquots (20 µl) were then loaded onto 10% Tris-Glycine polyacrylamide gel: final deposits of purified Opd are 115, 58.5, 28.5, 14.5 and 7.2 ng. Two samples of solubilized magnetosomes membrane proteins were prepared accordingly by mixing either 15 or 10 µl of solubilized magnetosome membrane proteins with 5 µl of SDS sample buffer (and 20 mM Hepes buffer (pH 8) add to a final volume of 20 µl when needed). After 5 mn boiling the two aliquots (20 µl) were onto the gel. For further calculations, one has to remember that the magnetosome suspension was diluted once for solubilization (320 µl in 400 µl final volume). After migration in Tris-Glycine buffer, 0.1% SDS, proteins were transferred on a nitrocellulose membrane using a Biorad® semi-dry transfer apparatus (0.25 A, 25 mn). The quantity of Opd immobilized onto the magnetosome surface was estimated by Western Blot analysis with antibodies raised against Opd. Immunization of one rabbit with 2 mg of purified Opd was made by Millegene® and the serum at J+69 after immunization collected. The nitrocellulose membrane was incubated overnight in TBST+5% skimmed milk at 10°C then washed 3 times in TBST (15, 5 and 5 mn). After incubation during 2 hours at room temperature with anti-Opd serum (1/50,000 in TBST), the membrane was washed 3 times in TBST (15, 5 and 5 mn) then incubated 1 hour with anti-rabbit IgG coupled to peroxidase (1/20,000 in TBST). After 3 washing steps (15, 5 and 5 mn), the chemiluminescence reaction was initiated by incubating the membrane 5 mn with the reagents. The chemiluminescent signal was measured in a G:Box image acquisition system (Syngene) and signal quantification carried out with the GeneTools analysis software from Syngene. The chemiluminescent signal is displayed in Fig. 3A and the calibration curve in Fig. 3B.

### TEM to visualize magnetosomes alignment

Aliquots of 10 µl either AMB-1 cells or magnetosomes suspension were spotted on formvar-carbon and after 2 min the excess liquid was drained. Unstained grids were observed using a Zeiss EM9 transmission electronic microscope at 80 kV.
